# Hepcidin and *HFE* Polymorphisms and Ferritin Level in β-Thalassemia Major

**Published:** 2019-01-01

**Authors:** Kiavash Fekri, Negar Asle Rasouli, Seyyed Abdolhossein Tavallai Zavareh, Milad Jalil, Fahimeh Moradi, Maryam Hosseinpour, Hossein Teimori

**Affiliations:** 1 Cancer Research Center, Shahrekord University of Medical Sciences, Shahrekord, Iran; 2 Medical Biotechnology, Cellular and Molecular Research Center, Basic Health Sciences Institute, Shahrekord University of Medical Sciences, Shahrekord, Iran; 3 Department of Pediatrics, School of Medicine, Shahrekord University of Medical Sciences, Shahrekord, Iran; 4 Molecular Genetics, Tehran Medical Branch, Islamic Azad University, Tehran, Iran; 5 Human Genetics, Cellular and Molecular Research Center, Basic Health Sciences Institute, Shahrekord University of Medical Sciences, Shahrekord, Iran; 6 Molecular Genetics, Cellular and Molecular Research Center, Basic Health Sciences Institute, Shahrekord University of Medical Sciences, Shahrekord, Iran; 7 Medical Genetics, Cellular and Molecular Research Center, Basic Health Sciences Institute, Shahrekord University of Medical Sciences, Shahrekord, Iran

**Keywords:** Ferritin, Hepcidin, HFE* Iron overload, Thalassemia

## Abstract

**Background:** Thalassemia patients need repeated transfusion that lead to increased blood ferritin level and iron overload in the heart and liver. Because the roles of hepcidin antimicrobial peptide (HAMP) and hemocromatosis protein (HFE) in iron metabolism have been confirmed, this study investigated the effects of these gene's polymorphisms on blood ferritin levels and iron overload in the heart and liver in patients with beta thalassemia major

**Materials and Methods: **This cross-sectional study was conducted on 91 patients referring to the Hajar Hospital in Shahrekord, Iran in 2015. After the blood samples were collected, the ferritin levels were measured, DNA was extracted from the blood cells, and the types of polymorphisms were determined using PCR-RFLP. Data of MRI T2^* ^in the heart and liver were drawn from the patients' medical files. Data analysis was conducted by *t*-test, chi-square test, Fisher's exact test, and Pearson correlation coefficient.

**Results: **There was no significant correlation between blood ferritin level and c.-582 A>G polymorphisms of hepcidin gene (p=0.58), and H63D of HFE gene (*p*=0.818). In addition, there was no significant association between the polymorphisms and heart and liver MRI, but there was a significant association between blood ferritin level and qualitative heart and liver MRI (*r*=-0.34, *p*=0.035 and *r*=-0.001, *p*=0.609, respectively).

**Conclusion: **In patients with β-thalassemia major, the presence of c.-582A>G HAMP and H63D HFE polymorphisms is not effective on blood ferritin level and iron overload in the heart and liver in the studied region.

## Introduction

 β-Thalassemia is the most common inherited anemic disorder in Iran; this disorder is caused by mutations in β globin gene. β-Thalassemia-associated anemia occurs due to decreased β globin chain synthesis, increased degradation of available red blood cells (RBC) and short RBC survival (^1^^-^^2^). This disorder is associated with various secondary complications such as extramedullary hematopoiesis, splenomegaly, iron overload, and blood-borne infections. The main cause of mortality in patients with transfusion-dependent thalassemia is iron overload-associated cardiomyopathy^2^. Ferritin is the main form of iron reserve in the body which is found mainly in reticuloendothelial system, hepatocytes, spleen, and bone marrow. Besides that, small amounts of ferritin can also be found in circulating plasma^3^. Ferritin concentration represents iron reserves in the body^4^. Iron metabolism in the body is regulated by several genes, including hepcidin antimicrobial peptide (HAMP) and human hemocromatosis (HFE, high Iron Fe) genes^2^^,^^5^. 

Hepcidin that is mainly synthesized by hepatocytes plays a role in the control of iron absorption and recycling. Hepcidin regulation in hepatocytes is affected by certain factors such as the amount of available systemic iron, hepatic iron reserve, erythropoietic activities, hypoxia, inflammation and infection. The regulation of hepcidin expression is a complex process that requires coordination among several proteins. Hepcidin dysregulation contributes to developing many diseases such as the anemia of chronic disease, iron-refractory iron deficiency anemia, cancer, hereditary hemochromatosis, and ineffective erythropoiesis such as β-thalassemia. Iron regulation by hepcidin is clinically important in anemia due to iron overload that occurs in β-thalassemia^2^. 

Hereditary hemocromatosis is an iron overload disorder that causes damage to several organs and is often due to HFE disfunction^5^. The HFE plays its most prominent role in the regulation of cell iron absorption that is accomplished by binding to TFRC (Transferrin Receptor). When cell has adequate amounts of iron, it competitively binds to TFR and prevents iron absorption via endocytosis^6^. To date, many polymorphisms of the HFE have been identified out of which H63D and C282Y have been reported to be comparatively more frequent^7^. H63D polymorphism entails replacement of aspartic acid 63 by histidine due to nonsynonymous substitution of cytosine with guanine at nucleotide 187 of the HFE gene^8^. Results indicate that the presence of H63D causes increase in serum ferritin, compared to the wild type. β-Thalassemia is generally characterized by potential development of iron overload and exacerbated with coinheritance of H63D polymorphism^9^. 

Because c.-582 A>G and p.H63D polymorphisms are two of the most common polymorphisms in hepcidin and HFE genes, Our aim is to investigate the association between the presence of these polymorphisms and blood ferritin level in high altitude and low pressure region in Iran. 

## MATERIALS AND METHODS

 This cross-sectional study was conducted on 91 patients referring to the Thalassemia Wards of Hajar Hospital in Shahrekord and Shohada hospital in Lordegan in 2015. First, the protocol of the study was approved by the Ethics Committee of the Shahrekord University of Medical Sciences (approval code no: 92-7-19). Sample size was determined approximately 91 by the formula below:

n = z^2^p(1-p) / d^z^

where z=1.96, p=0.35, and d=0.1. The inclusion criteria were history of at least 10 transfusions and having taken chelator for at least three months, and the exclusion criteria was lack of volunteering to participate in the study. The patients were ensured that their information would be kept private. The patients also provided informed consent for taking their blood samples before sample collection and as the blood samples were being taken, the patients filled out checklists of individual characteristics including​gender, age, place of residence, and medical history. 

None of the patients had symptoms of active hepatic disease and recent or current infection or inflammatory disease that may affect the expression of hepcidin and the level of ferritin. The patients' information such as the type of chelator therapy and the results of MRI T2^* ^assessments were obtained from their medical files. Ejection fraction (EF) was measured using standard CMR sequence and Quantification of iron deposition were categorized based on Table 1^19^. The blood samples were sent to the Shahrekord Central Laboratory to conduct serological examinations and measure blood ferritin levels by enzyme-linked radioimmunoassay method (Monobind Kit, USA). 

**Table 1:** Quantification of iron deposition in cardiac muscles and lever^19^

**Myocardial loading**	Myocardial T2*(ms)
Normal	>20
Mild	14-20
Moderate	10-14
Severe	<10
Hepatic loading	Hepatic T2*(ms)
Normal	>6.3
Mild	6.3-2.8
Moderate	1.4-2.7

**Preparation of genomic templates and PCR amplification**

The genomic DNA of the peripheral blood samples was extracted by phenol-chloroform extraction method (10). DNA Concentration was measured by using pure ethanol and salt at -20°C. DNA precipitation was suspended in 100 μL of TE buffer (Tris-Hcl10 mM, EDTA 1 mM) and the final solution was stored at -20°C till later use.

 The total volume of PCR solution was 50 μL. Table 2 shows the primers that were designed by Primer3 software for PCR amplifications using a gradient Gene Atlas G02 gradient thermal cycler (ASTEC, Fukuoka, Japan). PCR cycle to amplify a 179 bp fragment containing c.-582A>G polymorphism of hepcidin consisted of one cycle at 95°C for 10 min (initial denaturation), 35 cycles of 95°C for 20 s, 60°C for 20 s, 72°C for 20 s, and a final cycle of extension at 72°C for 5 min. 

PCR cycle to amplify 208 bp amplicon containing HFE H36D was similar to that of hepcidin project except that the annealing temperature which was at 58°C for 20s. 

**PCR-RFLP analysis and sequencing of DNA fragments**

To conduct enzymatic digestion of the PCR product with each enzyme, 1 µL of the enzyme (fermentas) and 2 µL of the buffer were added to 10µL of the sample DNA and then distilled water was added to the resulting solution to a final volume of 20µL. The vial containing the solution for enzymatic digestion alongside the enzymes was left under temperatures specified for each enzyme (Table 2) for 16 hr to allow complete enzymatic digestion. Then, the products of enzymatic digestion were examined by electrophoresis using acrylamide gel 8%. Fermentase DNA ladder (#SM 0321 bp Gene Ruler TM 50) were used to quantitatively analyze target DNA. The shifted bands were sequenced by Pishgam Co. (Iran) for final confirmation (Table 2).

**Table 2:** Primers and enzymes used in PCR-RFLP

**Gene**	**Region**	**Amplicon size**	**Primer pairs**	**Enzyme**	**Incubation** **Temperature**
Hepcidin	Promoter	179bp	F: GTGCTGGGCCATATTACTGCT R: CACGTGCATAGGTTCTGGCA	BstuI	60 °C
HFE	Exon 2	208bp	F: ACATGGTTAAGGCCTGTTGC R: GCCACATCTGGCTTGAAATT	BclI	50 °C

**Table 3:** Association between blood ferritin level and genotypes according to t-test

		**Genotype**	**Mean level of ferritin**	**P- Value**
H63D Polymorphism		AA	167±1891	0.818
		**AG**	**334** **±** **1975**	
C.-582A>G Polymorphism		**AA**	**1980** **±** **186**	0.55
		**AG**	**249** **±** **1760**	

**Statistical analysis**

Data analysis was conducted by *t*-test, chi-square test, Fisher's exact test, and Pearson correlation coefficient in the SPSS version 19. *P*< 0.05 was considered significance level. 

## Results

**Figure 1:**
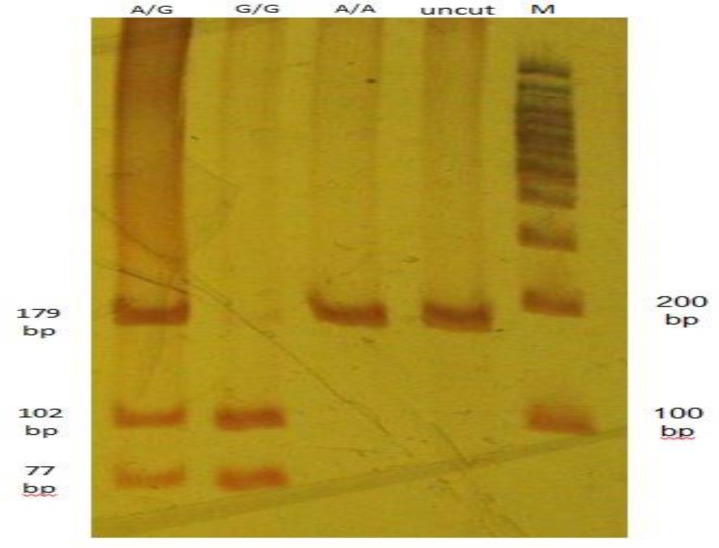
Detection of the Hepcidin c-582 A>G polymorphism by PCR-RFLP with Hepcidin promoter primers and digested with restriction enzyme Bstu1. Lane M: DNA marker (100bp). Lane uncut: PCR product without digestion. Lane A/A: homozygote for wild type allele that doesn’t be digested (179 bp). Lane G/G: homozygote for c-582A>G variant showing digested bands (102bp and 77bp). Lane A/G: heterozygotes for c-582A>G showing normal undigested band (179bp) as well as the digested c-582A>G variant (102 pb and 77bp).

**Figure 2:**
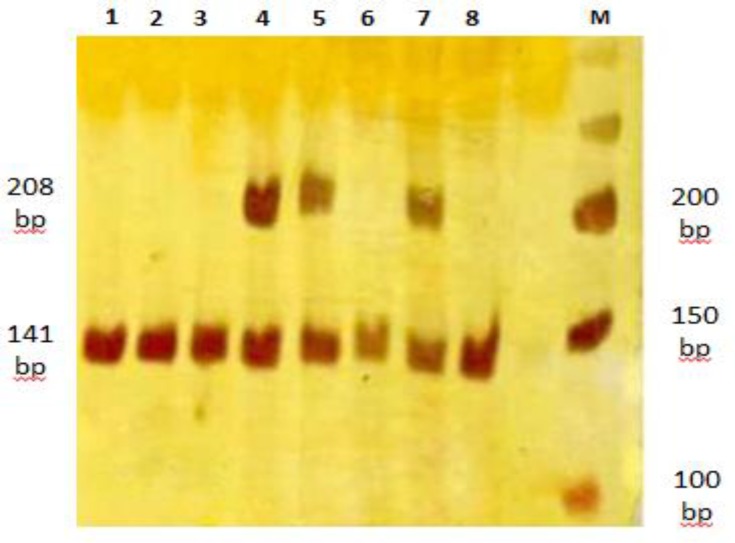
PCR-RFLP by H63D primers and Bcl1 as restriction enzyme. Lane M: DNA marker (50 bp). Lanes 4, 5, 7: homozygote for wild allele cut in to a 141bp and 67 bp fragment (not visible on gel). Lanes 4,5 and 7: heterozygotes for H63D variant showing normal digested bands (141 and 67bp) as well as the undigested amplified fragment (208 bp).

**Figure 3:**
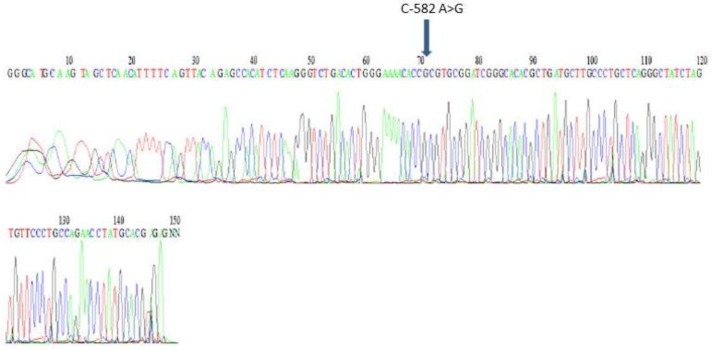
Sequencing result of a patient with c-582A>G polymorphism in promoter of HAMP gene in the position of 1490. Arrow shows the position of the polymorphism.

 In this study, 42% of the participants were female. The mean age of the participants was 19±7.5 years and the mean weight was 42±13.6 kg. RFLP analysis was performed for all patients (Figures 1 and 2). Also, sequencing were confirmed the polymorphisms (Figure3). Homozygosity for both polymorphisms (GG) was very rare. The types, frequency and percentages of used chelator and the genotypes are shown in Table 4. 

**Table4:** The frequency of chelators and genotypes of patients

		**Frequency**	**Percentage**
	Desferal	59	%64.3
	Deferiprone	9	%10.7
	Deferasirox	1	%1.2
Chelators	Desferal and Deferiprone	18	%1.9
	Desferal and Deferasirox	2	%2.4
	Deferiprone and Deferasirox	2	%2.4
	AA	65	%71.4
C.-582 A>G polymorphism	AG	25	%27.4
	GG	1	%1.4
H63D polymorphism	AA	71	%78
	AG	20	%22
Both polymorphisms	AG + AG	5	%5.49

## Discussion

Mean cardiac MRI (milisecond) was higher in patients with AA polymorphism than in those with AG polymorphism in the HAMP gene, representing decreased precipitation of cardiac iron in the AA polymorphism. In contrast, mean liver MRI (ms) was lower in patients with AA polymorphism than in those with AG polymorphism representing increased liver iron concentration in the AA polymorphism. Therefore, changes in the heart and liver were different. Heart MRI was normal in over half of the patients while liver MRI was normal only in 1/3 of them. Chi-square test indicated that there was no significant association between polymorphism and heart MRI (*P*=0.56). In patients with AG polymorphism, qualitative heart MRI was normal in 66% of them, mild to moderate in 34%, and severe in 0%. According to Spearman correlation coefficients, there was an inverse, significant correlation between blood ferritin level and quantitative heart and liver MRI (*r*=-0.34, *p*=0.035 and *r*=-0.001, *p*=0.609, respectively). 

In this study, we did not observe any significant association between blood ferritin level and the c.-582 A>G polymorphism in the patients that regularly consumed chelator, but a difference in blood ferritin level between the two genotypes was noted. This indicates that the polymorphism may be effective on the function of the HAMP promoter, which can be overwhelmed by regular consumption of chelators (environmental effect on the gene). However, this difference in ferritin level can be due to difference in the function of hepcidin protein, which was not investigated in the current study, and also the effect of other factors on increase in iron overload. A study on HAMP function in healthy people suggested that due to the key role of hepcidin in iron metabolism, different incidence of the hepcidin gene caused phenotypic differences among the patients. Consistent with our study, a study showed that no difference was observed in the ferritin level, iron, and transferrin levels in c.-582A > G. However, this difference can be attributed to the polymorphism role in the gene expression level, leading to different gene expression levels ^11^. 

Environmental conditions can also affect the hepcidin gene expression, including the type of nutrition and place of residence. For example, the people living in the highlands have lower hepcidin level compared to those living in the regions of lower altitiudes due to hypoxic conditions^18^.

Andreani et al.^12^ reported that the c.-582A>G polymorphism caused an increase in ferritin, which is inconsistent with our study. To explain this inconsistency, we can argue that our participants used iron chelators more regularly because the mean ferritin level in patients in the study of Andreani et al.^12^ was significantly higher than that in our participants. Besides that, given the lack of a significant association between the type of chelator and ferritin level, the type of chelator cannot be considered a confounding factor for ferritin level. Another explanation can be related to oxygen pressure in the studied region that can affect hepcidin gene expression in our participants because of the high altitude of Chaharmahal va Bakhtiari province. 

Regarding H36D polymorphism, the results of Andreani et al.^12^ are consistent with our​ study, because they also observed no significant difference in ferritin level between the two groups^12^. Heterozygosity can cause incomplete gene permeability^13^. In our study, people with hepcidin gene genotype AA had lower levels of ferritin level and higher iron precipitation in the liver. Hepcidin gene AG polymorphism in the current study was found to cause an increase the iron precipitation in the heart and a decrease in the iron precipitation in the liver, but the difference was not statistically significant. This evidence indicates that study of ferritin level alone cannot show the involvement of certain organs such as heart and liver. 

In our study, mean ferritin level was 2344±401 mg in the splenectomized people that is markedly different compared to 1803±143 mg in people without splenectomy; however, the difference was not statistically significant. In addition, endocrinopathies were associated with splenectomy, which is possibly due to higher levels of ferritin in the splenectomized patients. The findings of the present study imply that the presence of G nucleotide substitution in the patients leads to increased ferritin level as the marker of iron overload probably due to change in the transcriptional box in the HAMP promoter that causes change in gene translation factors. Our study also showed that the blood ferritin levels were not different in patients with β-thalassemia major with and without the polymorphisms, which is in agreement with the studies of Garewal et al., L´opez-Escribano et al., and Mellouli et al.^14^^-^^16^. 

A study to investigate the effects of HFE genotypes on iron markers in β-thalassemic carriers indicated that β-thalassemia was generally characterized by potential development of iron overload and tended to exacerbate with coinheritance of H36D polymorphism even in the heterozygous cases^17^. This inconsistency in our findings and that study can be due to the difference in environmental factors or genetic factors that lead to incomplete permeability of H63D genotype. Because the sample size of our study was small, studies with larger sample size should be conducted to further elucidate the role of these polymorphisms in iron accumulation in the heart. 

## CONCLUSION

 Blood ferritin levels were not different in patients with β-thalassemia major with and without the H63D and c.-582 A>G polymorphisms. However, study of these polymorphisms and their association with other factors, including environmental factor, can help explain increased blood ferritin level in patients with β-thalassemia major.
